# Psychiatric and Medical Management of Marijuana Intoxication in the Emergency Department

**DOI:** 10.5811/westjem.2015.3.25284

**Published:** 2015-04-09

**Authors:** Quan M. Bui, Scott Simpson, Kimberly Nordstrom

**Affiliations:** University of Colorado School of Medicine, Department of Psychiatry, Aurora, Colorado. University of Colorado School of Medicine, Denver Health Medical Center, Department of Psychiatry, Denver, Colorado

## Abstract

We use a case report to describe the acute psychiatric and medical management of marijuana intoxication in the emergency setting. A 34-year-old woman presented with erratic, disruptive behavior and psychotic symptoms after recreational ingestion of edible cannabis. She was also found to have mild hypokalemia and QT interval prolongation. Psychiatric management of cannabis psychosis involves symptomatic treatment and maintenance of safety during detoxification. Acute medical complications of marijuana use are primarily cardiovascular and respiratory in nature; electrolyte and electrocardiogram monitoring is indicated. This patient’s psychosis, hypokalemia and prolonged QT_c_ interval resolved over two days with supportive treatment and minimal intervention in the emergency department. Patients with cannabis psychosis are at risk for further psychotic sequelae. Emergency providers may reduce this risk through appropriate diagnosis, acute treatment, and referral for outpatient care.

## INTRODUCTION

Already the most commonly used illicit drug in the United States, marijuana (or cannabis) is becoming more widely used and more potent with expanded legalization.^1^–^3^ Legalization has also popularized “edible” forms of marijuana, including teas and food products. Although often portrayed as a harmless drug with potential therapeutic uses, marijuana has detrimental effects on brain development, psychiatric health (eg, psychosis, schizophrenia, depression and anxiety), lungs (eg, chronic bronchitis and lung cancer) and heart (eg, myocardial infarction and arrhythmias).^2^ Public perception of these risks decreases with legalization, and no guidelines exist to help patients gauge the personal safety of use.^4^,^5^ As emergency providers treat more patients with cannabis use disorders, they must educate patients about these chronic health risks and also manage the acute medical and psychiatric complications of marijuana intoxication.

To illustrate the management of acute complex marijuana intoxication and psychosis, we present a case of a woman requiring prolonged emergency department management after ingestion of edible tetrahydrocannabinol (THC), the active ingredient in marijuana.

## CASE REPORT

A 34-year-old woman with no significant psychiatric history presented to the emergency department (ED) with erratic and disruptive behavior. She broke into a neighbor’s home, requesting to “go to heaven.” She feared people were stealing from her and that “something bad” was going to happen. She reported insomnia, racing thoughts, and euphoria for the past week.

Upon arrival to the ED, her vital signs were temperature of 36.4°C, heart rate of 96bpm, blood pressure 148/111mmHg, and respiratory rate of 11. She was difficult to redirect and her mental status revealed a thin, “nervous,” well-groomed woman with a labile affect and pressured speech. The patient’s thought process was loose and disorganized with thought blocking. She was paranoid, grandiose, hyper-religious, and endorsed auditory hallucinations. She denied suicidal or homicidal ideation. Her attention and memory were considered impaired though not formally tested.

The patient admitted to using cannabis lip balm and consuming edible cannabis chocolate bars daily over the past week, most recently the day of presentation. She could not quantify her consumption. She believed her paranoia and insomnia onset coincided with her THC ingestion last week. The patient denied other recent substance or alcohol use. She denied any falls or history of traumatic brain injuries. A friend of the patient confirmed this history. Her other medications included propranolol 20mg twice a day for hypertension and infrequent sumatriptan as needed for migraines. Family history of mental illness was unknown since the patient was adopted.

For this presentation of acute psychosis, emergency medical providers conducted a comprehensive work-up to exclude organic etiologies of psychosis or concurrent medical morbidity. A basic metabolic panel was significant for a potassium level of 3.2mg/dL (reference range: 3.5–5.0); her electrocardiogram (EKG) demonstrated a prolonged QT_c_ of 508ms, a pulse of 86, and no U waves or T wave changes. A 9-carboxy-THC level was over 500ng/mL; her urine toxicology screen was negative for cocaine, amphetamines, benzodiazepines, and opioids. A B12 level was elevated at 1186pg/mL. Her complete blood count and a noncontrast head computerized tomography (CT) study were unremarkable.

The patient refused supplemental potassium, and it was thought that her EKG findings did not warrant emergent, forcible repletion. She also removed her intravenous line while agitated. She was placed in two-point soft restraints for her safety. After consultation with psychiatry, the patient was deemed medically appropriate for transfer to the ED’s psychiatric emergency service (PES) for further evaluation and treatment.

In the PES, the patient was hypersexual, hyperactive, and intrusive, entering other patients’ rooms and touching them. As she could not be safely re-directed, physical restraints were again ordered for the patient’s and others’ safety. Risperidone 0.5mg PO q6hr and lorazepam 1mg PO q6hr were ordered as needed for management of psychosis and anxiety; the patient required one dose of each during her PES stay.

Twenty-four hours after presentation, her psychotic symptoms and anxiety persisted: she suggested that her food was poisoned and asked whether she was African-American (though she was Caucasian). The patient claimed to have forgotten her father’s name, did not know where she was currently living, and was oriented only to person and place. She received her scheduled propranolol for hypertension and 40meq of oral potassium chloride (which she had earlier refused). Her consciousness and attention were intact.

Forty-eight hours after presentation, the patient’s paranoia and hallucinations improved dramatically. The patient was able to reflect on the unreality of her paranoia and “odd thoughts” of being African-American. With improved insight, she confirmed heavy use of multiple edible THC products in addition to frequent coffee and energy drink consumption, which she had difficulty quantifying. The patient was diagnosed with cannabis-induced psychotic disorder and severe marijuana use disorder; she was instructed to follow up with outpatient mental health to ensure resolution of her psychosis and begin substance abuse treatment.

## DISCUSSION

New-onset psychosis is a medical emergency with a broad differential.^6^ Signs and symptoms concerning for a medical etiology of psychiatric symptoms include abnormal vital signs, altered consciousness, or lack of prior psychiatric history in a patient over 40 years old.^7^ The acute onset of symptoms with marijuana use, high serum marijuana metabolite levels, and symptomatic resolution with detoxification suggest these symptoms were secondary to marijuana use.

Cannabis-induced psychotic disorder (“cannabis psychosis”) is diagnosed when psychotic symptoms persist beyond acute intoxication and may require clinical management.^8^ Psychiatric symptoms include paranoia, derealization, disorganized thinking, persecutory and grandiose delusions, hallucinations, and cognitive impairment. Patients pose a danger to others and themselves due to their altered sense of reality. Safe cannabis detoxification typically requires 24 hours, but sometimes longer for patients with unstable vital signs and persistent psychosis. Benzodiazepines are recommended for agitation related to stimulant intoxication – unless psychosis is present, in which case oral atypical antipsychotics are considered first-line.^9^

Cannabis blood levels reflect the extent and chronicity of marijuana use. A free THC level below 3ng/mL (μg/L) suggests occasional consumption (≤1 joint/week) while a concentration higher than 40ng/mL corresponds to heavy use (≥10 joints/month).^10^ Levels above 10ng/mL impair motor function, leading two states with legal recreational marijuana to establish the legal limit for driving at 5ng/mL. In clinical practice, measuring an inactive metabolite of THC, 9-carboxy THC, is preferred due to the rapid decrease in free serum THC levels.^11^ In a prior case report, oral cannabis-induced psychosis resolved within 24 hours after recorded serum THC levels below 20ng/mL, or 9-carboxy-THC levels below 50ng/mL; the authors suggested that oral administration may not achieve high serum THC levels.^12^ Our patient’s 9-carboxy-THC level over 500ng/mL demonstrates that oral administration can achieve high serum THC levels and suggests a dose-response relationship between serum metabolite levels and the severity of psychosis. Moreover, serum drug levels may anticipate a patient’s clinical course.

The medical risks of acute cannabis use are primarily cardiovascular in nature. THC enhances sympathetic tone, thereby increasing heart rate and blood pressure.^13^ Marijuana increases the risk of myocardial infarction within one hour of use, and cardiovascular events have been reported in otherwise healthy patients.^5^,^14^,^15^ A Norwegian autopsy study suspected THC-induced arrhythmias (including ventricular tachycardia and fibrillation) as the culprit in six patients who died suddenly.^15^,^16^ Electrocardiograms should be obtained for patients with severe cannabis intoxication; telemetry monitoring may be considered for patients with known cardiac pathology.

Electrolyte abnormalities reported in marijuana users contribute to this cardiac pathology. Chronic marijuana users have lower serum sodium and potassium than non-users.^17^ The heavy consumption of carbohydrates while intoxicated leads to an increase in serum insulin levels, driving potassium into cells and causing serum hypokalemia.^18^ This hypokalemia can produce reentrant arrhythmias by decreasing conductivity and increasing the resting membrane potential, duration of the action potential, and duration of the refractory period.^19^ EKG changes include the decrease in T-wave amplitude, presence of U waves and a prolonged QTc. This patient’s very high THC metabolite level, prolonged QTc, and hypokalemia increased her risk for an arrhythmia. The hypokalemia observed in this case was likely related to acute intracellular potassium shifts superimposed on chronic hypokalemia.

Clinicians must manage other, non-vascular risks of acute marijuana use. Respiratory symptoms include shortness of breath, wheezing, and even respiratory failure when marijuana has been smoked “wet” with phenylcyclidine or embalming fluid.^20^,^21^ Patients with pre-disposing genetic vulnerabilities may develop hypokalemic periodic paralysis.^18^ And, marijuana use correlates with fatal motor vehicle collisions – clinicians should educate patients and ensure a safe transportation plan on discharge.^22^

Patients with toxic ingestion must be screened for co-ingestion. The persistence and intensity of the patient’s symptoms warranted consideration of multiple involved substances. Co-ingestion may also be signaled by an abnormal osmolar or anion gap, positive urine toxicology screen, or QTc or QRS prolongation (Only QTc prolongation was present here).^23^,^24^ However, in many cases, the presence of co-ingestion may only be detected once the patient is able to provide a reliable history. In this case, an elevated B12 level was found on work up of the patient’s psychiatric symptoms and suspected to have been caused by energy drink consumption; only later did the patient confirm this suspicion. By its effects on mesolimbic dopamine activity, caffeine may precipitate psychosis, exacerbate chronic psychosis, or worsen affective lability and mood states.^25^–^28^ This patient’s high THC metabolite level and medical course are consistent with cannabis psychosis; however, we cannot exclude excessive caffeine use as a contributor to this presentation.

What is this patient’s prognosis? Marijuana correlates with the onset of psychosis in patients with schizophrenia and perhaps bipolar disorder as well.^29^–^32^ About half of patients with cannabis psychosis will later be diagnosed with a primary psychotic disorder.^8^,^33^ This high rate may reflect high rates of marijuana use among patients with schizophrenia. Younger age, greater frequency of marijuana use, family history of psychosis, trauma history, and schizotypal personality correlate with higher risk of a later diagnosis of primary psychosis.^8^ ED providers can mitigate the risk of psychopathology by addressing the patient’s substance use disorder. Safe detoxification is a primary goal and was accomplished here; brief interventions like motivational interviewing and referral for treatment in the ED may reduce use on discharge.^34^,^35^
